# Effect of passion on the athlete engagement of college students specializing in DanceSport: the mediating role of the DanceSport partnership

**DOI:** 10.3389/fpsyg.2024.1503279

**Published:** 2024-12-23

**Authors:** Huang Ziyou, Yuan Yuxin, Li Xiaofen

**Affiliations:** ^1^School of Physical Education, Anqing Normal University, Anqing, China; ^2^School of Arts, Beijing Sport University, Beijing, China

**Keywords:** athlete engagement, passion, DanceSport, partnership, mediation effect

## Abstract

**Objective:**

This research investigates the influence of passion on DanceSport engagement (DSE) among university students specializing in DanceSport, along with the mediating function of DanceSport partnership (DSP).

**Methods:**

A survey involving 1,029 participants was conducted using the Passion Scale, Athlete Engagement Questionnaire, and Chinese DanceSport Partnership Scale.

**Results:**

There were significant positive associations among passion, DSP, and DSE. The path coefficient revealed that passion had a 0.631 (*p* < 0.001) effect on DSE, a 0.548 (*p* < 0.001) effect on DSP, and DSP had a 0.217 (*p* < 0.001) effect on DSE. The direct impact of passion on DSE was 0.725, while the indirect impact was calculated at 0.137, resulting in a total effect of 0.862.

**Conclusion:**

Passion was identified as an internal motivator enhancing DSE, whereas DSP was found to be an external factor contributing to the increase in DSE levels. DSP played a partial mediating role in the influence of passion on DSE, the more harmonious the DSP, the higher the engagement level. In the future, by understanding the current status and trends of college students’ passion, DSE, and DSP, coaches can timely adjust course content to enhance engagement levels and sports performance by exchanging partners and changing cooperation forms.

## 1 Introduction

DanceSport is a kind of sport with a normative and procedural nature that is performed by male and female couples, is within the scope of defined music and rhythms, and demonstrates artistic expression with physical, technical skills (Li, 2016). For college students specializing in DanceSport, the construction of a normative and procedural technical system and the shaping of artistic style cannot be separated from active and long-lasting movement engagement in the practice of DanceSport.

Engagement in sport is a positive and lasting psychological state characterized by self-confidence, dedication, vitality and enthusiasm (Lonsdale et al., 2007), which is an important factor in stimulating athletes’ positive qualities, such as optimism, resilience, and creativity; enhancing their athletic abilities; and pursuing excellence in academics and competition results (Zhang, 2012). In this study, DanceSport engagement (DSE) was defined as the degree of students’ participation in DanceSport activities. Factors such as cognitive understanding of the sport, emotional involvement, experiential status, persistence, and the use of metacognitive strategies by individuals (Dan and Liu, 2023) can predict their identification with the sport and their motivation to participate in its practice (Lodah and Kejner, 1965). This suggests that a higher level of DSE and greater satisfaction with DanceSport are associated with a stronger intention to continue practicing (Vecina et al., 2012).

In the context of China’s “14th Five-Year Plan” for a strong sports nation (General Administration of Sport of China, 2021), which has achieved important results in various fields and projects, guiding college students’ positive cognitive, emotional, and behavioral inputs to DanceSport is related to the quality of competitive talent cultivation and the development of sports projects. Therefore, examining the influencing factors and mechanisms of students’ DSE is an important issue that urgently needs to be addressed for the cultivation of talent in colleges and universities and the development of DanceSport in China.

In recent years, psychologists have highlighted that passion serves as an internal motivator for sustaining and enhancing sport engagement (Parastatidou et al., 2012; Carbonneau et al., 2010). Passion is defined as a strong inclination to dedicate time and effort to an activity that one finds enjoyable and perceives as significant. This inclination is internalized into either harmonious passion or obsessive passion, Harmonious passion allows individuals to willingly choose to engage in or withdraw from an activity with a sense of control, whereas obsessive passion involves an uncontrollable urge to participate or refrain from the activity (Vallerand et al., 2003). Studies indicate that passion stimulates mastery goals through mechanisms that encourage deliberate practice, which in turn positively predicts objective performance (Vallerand et al., 2008). This suggests that passion can motivate individuals to remain committed to activities that foster intentional practice, thereby creating optimal opportunities for learning, skill acquisition, and achieving excellence. Passion not only objectively represents the number of times an individual regularly engages in exercise (Carbonneau et al., 2010) but is also a predictive source of the quality of inputs during strenuous exercise (Parastatidou et al., 2012). Harmonious passion significantly predicts engagement (Oh, 2018). A latest study showed that the highest levels of engagement were associated with high harmonious passion (Schellenberg and Lötscher, 2024). These information illustrates that passion is an endogenous motivator for the development of engagement.

Psychologists have reported that interpersonal relationships within the sphere of activity have a mediating effect on the influence of passion on behavior (Kim et al., 2019). The dualistic model of passion suggests that passion can influence the relationships of those interacting with the individual within the context of the activity, whether it is a cooperator of equal status (e.g., athlete–athlete relationship; Stenseng et al., 2015) or a manager of higher status (e.g., coach–athlete relationship; Jowett et al., 2013). DanceSport partnership (DSP), as a relationship of equal status in DanceSport, refers to the interpersonal connection formed by pairs of partners throughout the training, competition, and life process with the goal of obtaining excellent performance, which is composed of three distinct and interrelated components representing obligatory instrumental ties, expressive ties, and interpersonal perception (Liu et al., 2022). First, passion predicts the quality of the dance partnership. Previous studies have shown that passion has a significant effect on partner relationships, with harmonious passion not only positively predicting individuals’ attitudes toward their partners, understanding their partners, and promoting healthy partner relationships (Kim and Park, 2015) but also strengthening the sense of trust between partners, forming a healthy and harmonious relationship between partners, and thus obtaining the benefit of the judges’ and audience’s reactions (Yang, 2016). Second, partnering serves as an external factor influencing movement engagement for college students specializing in sport dance. According to self-determination theory, activities are motivated by both extrinsic and intrinsic factors to achieve more favorable outcomes (Vallerand, 2021). Previous empirical research has shown that a dancer’s partnership acts as an external motivator for exercise engagement, and enhancing trust between dance partners and establishing a harmonious partnership can lead to a higher level of commitment to the activity (Kim and Park, 2019). Dance partners can reinforce the effects of movement engagement by fostering a sense of connection and cohesion with one another (Jim and In, 2006). Notably, a study utilizing a subject-object interdependence model demonstrated that partners with strong emotional connections were able to enhance not only their own sport engagement but also the quality of their partner’s engagement (Liu et al., 2024), suggesting that dance partnering significantly contributes as an external factor to increased movement engagement.

From the successful experience of the attribution and improvement mechanism of sports engagement (Wang et al., 2014), it is clear that the path to promote the enhancement of sports engagement should integrate the interactive effects of endogenous and exogenous factors to consolidate the scientific effectiveness of the improvement strategy. Moreover, the typical characteristics of DanceSport and the beauty of movement come from partner cooperation (Budnik-Przybylska et al., 2015; Chae and Koh, 2012). An excellent and suitable partner is a decisive factor in achieving the goals of learning, training, and competing in DanceSport. Many champion pairs have disbanded due to conflicts, afterwards, without finding a suitable partner, they lose their passion for DanceSport, stop competing, and even give up dancing. Therefore, solving problems related to DanceSport should always take into account the important influence of partnering (Pilewska et al., 2013), which is a contingent need for the theoretical deepening of DanceSport and a real requirement for the development of project practice. However, studies in the field of sport psychology have focused more on the effects and causes of passion on performance (Bill and Philippe, 2023; Vallerand et al., 2008), with very little attention given to DanceSport psychology (Oh, 2018) and, even more importantly, the important role of the partner relationship in this area. No further empirical studies demonstrated whether DanceSport partnerships mediate the effect of passion on athlete engagement. Therefore, this study, starting from the DSP, aims not only to fill the gap in the mechanism of passion influencing DSE but also to provide a new perspective for researching the theoretical development of DanceSport and promoting sustained DSE. Based on this, we aimed to study the following questions: (1) Does passion and DSP affect DSE? (2) Does DSP have a mediating effect?

## 2 Methods

The present study used a cross-sectional design, and before all the participants completed the questionnaire, they were informed of the purpose of this study and pertinent ethical considerations (including confidentiality, anonymity, the right to withdraw, and data protection). The study was conducted with the assurance that all participants were informed and willing to do so.

### 2.1 Participants

To ensure the representativeness of the sample, we employed a combination of judgment sampling and convenience sampling, and chosed students majoring in DanceSport from Beijing Sport University, Beijing Dance Academy, Chengdu Sport University, Wuhan Sport University, Guangzhou Sport University, Nanjing Sport University, Xi’an Sport University, Shenyang Sport University, Jilin Sport University, Anhui Normal University, Hubei Normal University, Anqing Normal University, and Gannan Normal University as our sample. The selection of these universities was based on the establishment of DanceSport programs and participation in competitions, as well as the students’ partnership experience. (1) The sports institutions have representative programs, thus almost all sports colleges are included. (2) There are relatively few normal universities offering sports dance programs, and even fewer participating in sports dance competitions, hence convenience sampling was adopted. (3) Beijing Dance Academy is an art college that has consistently participated in sports dance competitions.

In the present study, questionnaires were distributed and collected with the assistance of teachers at the chosen universities. We used the uniform instruction of the questionnaire, and randomly changed the order of the items in the scale. A total of 1,122 questionnaires were distributed and collected, with a recovery rate of 100%. The judge used the following inclusion criteria: having partner experience. Additionally, questionnaires with missing information, regular answers, or paradoxical choices were excluded. The final retained 1,029 valid questionnaires, the sample situation is shown in Table 1.

**Table 1:** Demographic information of the participants (*n* = 1,029).

Variable	Classification	Frequency	Percent (%)
Sex	Man	325	31.6
	Female	704	68.4
Education background	Undergraduates	912	88.6
	Postgraduates	117	11.4
Training time	1 ~ 5 years	315	30.6
	6 ~ 10 years	411	39.9
	>10 years	303	29.4
Partner time	1 ~ 6 months	392	38.1
	7 ~ 12 months	204	19.8
	13 ~ 24 months	178	17.3
	>24 months	255	24.8

## 3 Measurements

### 3.1 Passion scale

The passion scale designed by Vallerand et al. (2003), which is composed of two dimensions, namely, obsessive passion (OP) and harmonious passion (HP), with a total of 12 items, was utilized to determine the level of passion of the participants in the present study. To better contextualize the present study, we added the theme “DanceSport,” such as “I can fully demonstrate my talent by engaging in DanceSport” in the HP dimension and “Sometimes I feel that DanceSport has completely controlled my behavior and life” in the OP dimension. The passion scale is based on a 5-point Likert scale, which ranges from “strongly disagree” (1 point) to “strongly agree” (5 points). The score of the scale is positively related to passion. The scale had been translated and tested in China with good reliability and validity (Qingbao, 2018). In this study, the passion scale has been shown to have high reliability and validity (Cronbach’s *α* = 0.91, χ^2^/df = 1.43, RMSEA = 0.02, GFI = 0.98, AGFI = 0.97, NFI = 0.98).

### 3.2 Partnership scale-DanceSport couples

We used the Partnership Scale-DanceSport Couples (PS-DSC) (Liu et al., 2023a) to determine the participants’ partnership in the present study. The PS-DSC consists of three dimensions, namely, obligatory instrumental ties, expressive ties, and interpersonal perception, with a total of 12 items. The scale is based on a 5-point Likert scale ranging from “strongly disagree” to “strongly agree.” The score of the scale is positively related to the degree of partnership. In this study, the PS-DSC was proven to have high reliability and validity (Cronbach’s *α* = 0.95, χ^2^/df = 1.77, RMSEA = 0.03, GFI = 0.99, AGFI = 0.98, NFI = 0.99).

### 3.3 Athlete engagement questionnaire

We adopted the athlete engagement questionnaire designed by Lonsdale to evaluate DSE in the present study (Lonsdale et al., 2007). The scale, which includes four dimensions (confidence, dedication, vigor, and enthusiasm), consists of 16 items and is based on a 5-point Likert scale ranging from “never” (1 point) to “always” (5 points). A higher score indicates greater engagement of the athletes. The scale had been translated and tested in China with good reliability and validity (Wang et al., 2014). In this study, the scale was shown to have high reliability and validity (Cronbach’s α = 0.97, χ^2^/df = 2.17, RMSEA = 0.03, GFI = 0.99, AGFI = 0.98, NFI = 0.99).

### 3.4 Analysis strategy

All the data were processed and analyzed using SPSS 27.0 and AMOS 28.0. After we screened all the data obtained from the questionnaire, SPSS 27.0 was used to screen all the data obtained from the questionnaire, conduct descriptive statistics, and perform correlation analysis. We subsequently utilized AMOS 28.0 to complete the confirmatory factor analysis, build structural equation model plots, test the goodness of fit of the model, perform path tests, and assess the bootstrap mediation effect.

## 4 Results

### 4.1 Common method bias

We used the untested single-method latent factor approach to control for common method bias because many psychologists believe that the Harman one-way approach to detecting common method bias is problematic (Tang and Wen, 2020; Wen et al., 2018; Xiong et al., 2012). We constructed validation factor models 1 and 2, which contained the method factor separately, and compared the differences in the main fit indices of the two models. The results showed that in Model 1, χ^2^/df = 1.45, RRMSEA = 0.02, AGFI = 0.97, and TLI = 0.99. In Model 2, χ^2^/df = 1.46, RRMSEA = 0.02, AGFI = 0.97, TLI = 0.98. ▵χ^2^/df = 0.01, ▵RMSEA = 0.00, ▵AGFI = 0.00, ▵TLI = 0.01. The change in each fitting index was less than 0.03, indicating that no serious common method bias existed.

### 4.2 Correlation of each variable

We used the Pearson correlation coefficient to determine the relationships between passion, DSP, and DSE.

The results above revealed that, among the students specializing in DanceSport, (1) a significant positive correlation between passion and DSE existed (*r =* 0.672, *p* < 0.01), which indicated that the greater the degree of passion the students had, the more they engaged in DanceSport. (2) There was a significant positive correlation between passion and DSP (*r =* 0.481, *p* < 0.01), which indicated that the more passion the students had, the better the partnership they had. (3) A significant positive correlation between DSP and DSE existed (*r =* 0.540, *p* < 0.01), indicating that the better partnership participants had, the more they engaged in DanceSport (Table 2).

**Table 2:** Mean, Standard deviation, and Pearson correlation coefficient of each variable (*n* = 1,029).

Variables	*M*	SD	Passion	DSE	DSP
Passion	3.879	0.651	1.000		
DSE	3.896	0.717	0.672^**^	1.000	
DSP	3.551	0.817	0.481^**^	0.540^**^	1.000

** indicates *p* < 0.01. DSE, DanceSport engagement; DSP, DanceSport partnership.

### 4.3 Mediating effect of DSP between passion and DSE

**Figure 1:**
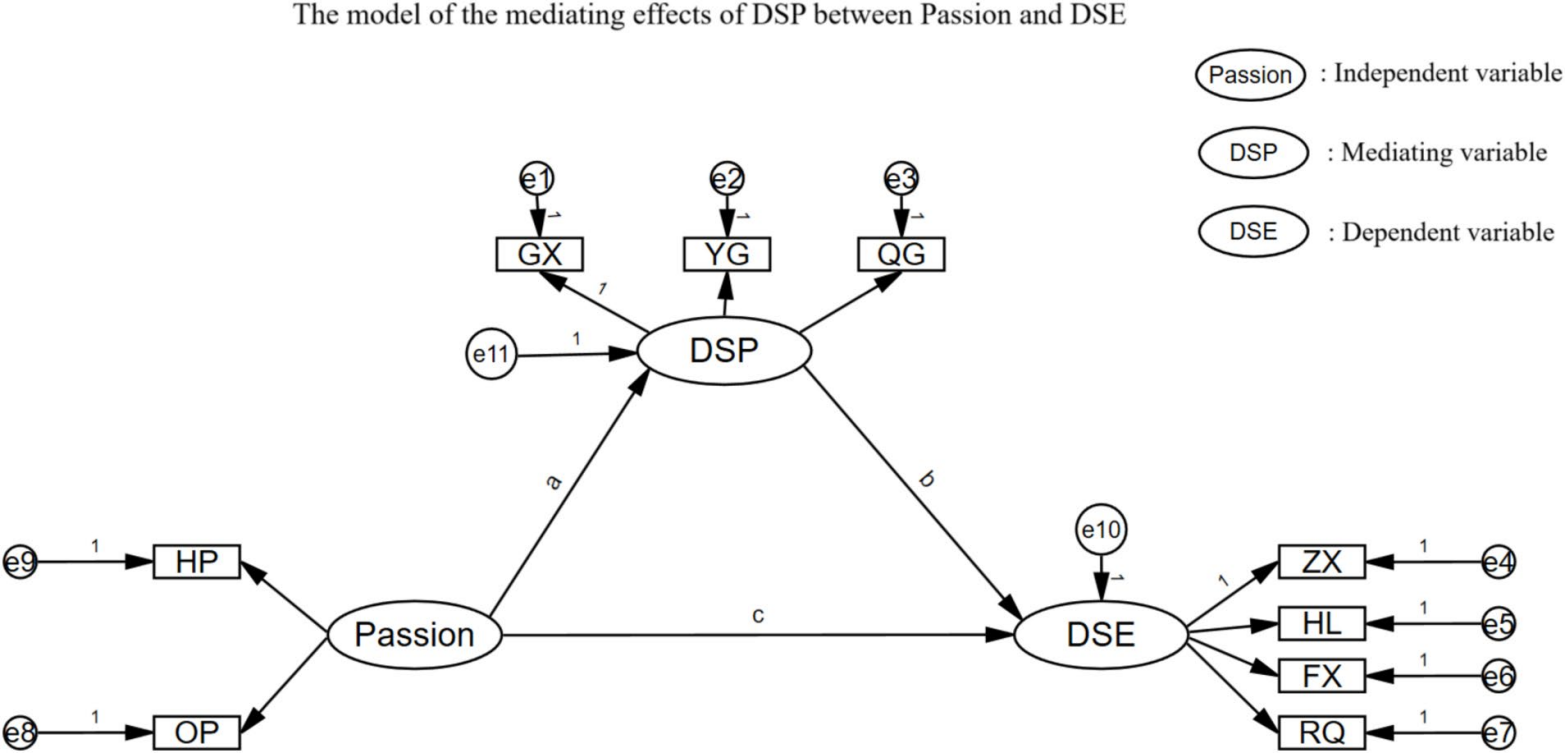
The model of the mediating effects of DSP between Passion and DSE.

Amos 28.0 was utilized to analyze the mediating effect of DSP through structural equation modeling, using the mean scores of passion, DSP, and DSE dimensions as latent and exogenous variables (Figure 1). According to the results, χ^2^/df = 1.33, RMSEA = 0.02, GFI = 0.99, AGFI = 0.97, and NFI = 0.99, indicating an acceptable fit of the model.

The path coefficients of the two-by-two relationships between passion, DSP, and DSE are shown in Table 3. (1) Passion had a significant positive effect on DSE (*β* = 0.631, *p* < 0.001), (2) passion had a significant positive effect on DSP (*β* = 0.548, *p* < 0.001), and (3) DSP had a significant positive effect on DSE (*β* = 0.217, *p* < 0.001).

**Table 3:** Results of path analysis of Passion, DSP, and DSE (*n* = 1,029).

Pathway			Estimate	SE	CR	*p*
Passion	→	DSE	0.631	0.040	18.123	***
Passion	→	DSP	0.548	0.045	15.300	***
DSP	→	DSE	0.217	0.028	7.071	***

***p < 0.001. DSE, DanceSport engagement; DSP, DanceSport partnership.

The bootstrap test was performed to test the mediation effect of DSP at the 95% CI via a bias-corrected method with 2000 repetitive samples. As Table 4 shows, the direct effect [Es = 0.725, 95% CI (0.633, 0.818)], indirect effect [Es = 0.137, 95% CI (0.089, 0.195)], and total effect [Es = 0.862, 95% CI (0.786, 0.939)] of passion for DSE were significant, indicating that DSP played a partial mediating role in the process of passion for DSE. The proportion of the mediating effect is 15.9%.

**Table 4:** Results of the mediating effects of DSP between Passion and DSE (*n* = 1,029).

Pathway	Effect	Estimate	95% CI		*p*
			Lower	Upper	
Passion→DSP → DSE	Direct	0.725	0.633	0.818	***
	Indirect	0.137	0.089	0.195	***
	Total	0.862	0.786	0.939	***

***p < 0.001. DSE, DanceSport engagement; DSP, DanceSport partnership.

## 5 Discussion

### 5.1 Direct effect of passion on DSE

The results of the correlation analysis and path analysis of the present study revealed that the greater the passion of the participants was, the more they engaged in DanceSport, which is consistent with previous opinions (Oh, 2018). The results prove the positive effect of passion on the development of DSEs (*β* = 0.631).

In the field of sport, passion, as a psychological and behavioral attitude of high-intensity positive emotions that can be experienced by individuals (Ho et al., 2011), is characterized by perceived internal motivation and appreciation of the value of sport (Pertula and Cardon Cameron, 2011), which predicts a high level of emotional state and motivational efficacy, motivating individuals to invest more time, energy and resources in the activities they love (Vallerand and Houlfort, 2019). As Slemp et al. reported, high levels of passion can stimulate more positive emotions in individuals, compelling them to continue investing cognitive resources, time, and energy, which significantly enhances engagement states (Slemp et al., 2021). It’s like many champions and competitors said, they love dance, every night before going to bed the brain will automatically review the dance actions and even dream of dancing. As the passion development theory posits passion is cultivated through a process in which individuals select activities, evaluate their significance, and incorporate them into their self-identity. It indicates that participants with a high level of passion have integrated DanceSport into their sense of self, which strengthens their dedication to the sport and enables them to remain committed despite challenges.

### 5.2 Direct effect of DSP on DSE

In the present study, our results showed that DSP was positively related to DSE, which is consistent with the results of previous studies (Kim and Park, 2019; Jim and In, 2006; Liu et al., 2024). DSP is an external facilitator that increases the level of athletic engagement of college students (*β* = 0.217). First, DanceSport is a form in which both couples share benefits and risks, responsibilities and obligations on the basis of mutual trust to achieve their goals. As Majoross noted, partnering is the foundation of the performance of DanceSport couples (Majoross et al., 2008). Couples who neglect partnering can negatively affect DSE and the quality of competitive performance (Davis et al., 2013). In DanceSport, achieving high-quality commitment and optimal performance is the ultimate goal. Therefore, to achieve this goal, couples establish an instrumental relationship to gain mutual benefit, which could further increase their DSE.

Second, couples can predict each other’s behavior, enhance tacit cooperation, and reduce conflicts through a series of harmonious relational interactions, such as communicating with partners, sharing emotions, and fulfilling training plans and commitments. As Kang suggested, interpersonal relationships originate from the process of individual perception of the interaction they are involved in Kang and Bodenhausen (2015). The DSE could be stably increased through the above process.

Ultimately, DSPs seem to be embedded in an idiosyncratic paradigm of “favors” in the Chinese context (Liu et al., 2022). Once college students enroll in school and choose to form a partnership with a good heterosexual dancer to achieve the best goals in DanceSport, the partnership takes on obligatory instrumental attributes. After a period of time, the perception of the DSP continues to grow and subsequently builds an emotional bond that favors the attributes of a “we” rather than an “I-him.” This emotional bond reduces utilitarianism and focuses more on the “human” aspect, which enhances the stability of the partner relationship and, to a certain extent, improves the quality, efficiency and satisfaction of the training (Jim and In, 2006). This suggests that a stable DSP is not only the basis of tacit cooperation but also the key to promoting DSE and even improving the level of competition and sports performance.

### 5.3 Direct effect of passion on DSP

We found that passion is an antecedent element that promotes healthy DSPs (*β* = 0.548), which is consistent with the findings of previous studies (Yang, 2016). According to passion theory, passion influences the nature of the interpersonal relationship process in terms of positive and negative emotions. Individuals with high levels of passion are generally energetic and appealing, and they are more likely to form high-quality relationships (Philippe et al., 2010). Passionate individuals often experience positive emotional states and effectively convey their emotions to others, as emotions are inherently social (Frijda, 1994). According to the broaden-and-build theory, positive emotions enable individuals to connect more deeply with themselves and with others (Fredrickson, 2001). Consequently, DanceSport students who exhibit sustained passion are typically more energetic, maintain a positive emotional state, and are capable of forming healthy and positive partnerships through regular, optimistic communication and emotional sharing during dance activities with their partners.

Furthermore, the fast-paced and emotionally intense nature of dances in DanceSport, including sensual movements, close physical proximity, eye contact, and synchronized breathing during training and competitions, requires participants to sustain a high level of passion. This passion can be harnessed to foster healthy and harmonious relationships between dance partners (Liu et al., 2023b).

### 5.4 Mediating effect of DSP

The findings of the current mediation effect analysis revealed that DSP partially mediated the relationship between passion and DSE, suggesting that the influence of passion on DSE is both direct and indirect. Dancers with sustained passion for DanceSport improve their collaborative coordination with partners through prolonged communication and the sharing of dance experiences, thereby enhancing the stability of the partnership and increasing both partners’ engagement in DanceSport. Furthermore, maintaining a high level of passion during dancing activities strengthens partner cooperation, elevates the quality of performance, fosters the harmonious development of the partnership, and gradually increases the level of DSP for both partners, even over the long term. As a mediating factor, DSP further explains the mechanism of the transformation of passion into DSE among DanceSport students. As a Chinese champion once confessed, she has not met a suitable partner since her partner chose to leave the competition, although she want to enter the competition again, but the result is not ideal. Obviously, this underscores the critical role of DSP in the growth of DanceSport and the nurturing of talent.

In DanceSport, the partnership is a fundamental aspect. Dancers must first establish physical communication, followed by emotional and mental synchronization. However, this is a prolonged and dynamic process that can be easily influenced by differences in partners’ personal lives, education, and the surrounding environment. Therefore, if both partners enhance their communication and understanding, openly share their thoughts, and support the healthy development of their relationship, they will experience greater excitement and enjoyment during training and competitions, ultimately increasing their commitment to DanceSport.

## 6 Strengths and limitations

The overall results of this study should be considered in terms of strengths and limitations. In terms of strengths, (1) from the perspective of partner relationship, it explains the pathway of passion into engagement, and fills in the gap of the mechanism of passion influencing the engagement of DanceSport college students. (2) It provides new perspectives for the development of the theory of DanceSport program and the improvement of sustained engagement of DanceSport college student, and enriches the theory of the study of passion and partner relationship. In terms of limitations. (1) The measurements of passion, partner relationship, and athletic engagement of DanceSport college students were all self-reported, which may be biased by social expectations. (2) Measurements of passion, DSP, and DSE were obtained simultaneously. It is recommended that future research collect data from multiple time points for longitudinal analysis, and also utilize objective engagement measurements to overcome the limitations of self-reporting.

## 7 Conclusion

Frist, this study delves into the effect of passion on DanceSport engagement among university students specializing in DanceSport, along with the mediating function of DanceSport partnership. Our findings underscore that passion is an internal factor that promotes the development of the engagement of the students specialized in DanceSport, partnership is an external factor that improves the level of engagement of students specializing in DanceSport, the passion college students specialize in DanceSport not only directly affects their engagement but also indirectly affects it by acting on the partnership. Secondly, the results show that the engagement level of college students specialing DanceSport can be improved through two ways: (1) Improve the level of passion. Coaches can increase their passion for dancesport by providing social support in the form of positive encouragement, or by adjusting course content to increase their passion level, such as by setting up mock competitions. (2) To promote the harmonious development of partner relationship. On the one hand, DanceSport students can often communicate with their partners, reduce the gap, and form a good relationship between dance partners. On the other hand, the coach can adjust the training strategies such as exchanging partners, changing the form of cooperation, cognitive communication of dance partner relationship to promote the harmonious development of dance partner relationship. Finally, the results may provide theoretical references for future research on sport training and engagement from both psychological and dyadic cooperation perspectives in other sports with similar cooperative relationships, such as figure skating.

## 8 Future research directions

Our study shows that the passion and DSP of DanceSport students will affect their own DSE. But will individual passion and DSE affect partner’s passion and DSE level? How DSE levels are affected by different types of rows with different purposes, and whether teacher-student relationship and parental support affect DSE levels remains to be further studied.

## Data Availability

The original contributions presented in the study are included in the article/supplementary material, further inquiries can be directed to the corresponding author.
